# Plant Defensin γ-Thionin Induces MAPKs and Activates Histone Deacetylases in Bovine Mammary Epithelial Cells Infected With *Staphylococcus aureus*

**DOI:** 10.3389/fvets.2020.00390

**Published:** 2020-07-24

**Authors:** Marisol Báez-Magaña, Nayeli Alva-Murillo, Ivan Medina-Estrada, María Teresa Arceo-Martínez, Joel E. López-Meza, Alejandra Ochoa-Zarzosa

**Affiliations:** ^1^Centro Multidisciplinario de Estudios en Biotecnología, Facultad de Medicina Veterinaria y Zootecnia, Universidad Michoacana de San Nicolás de Hidalgo, Morelia, Mexico; ^2^Departamento de Biología, División de Ciencias Naturales y Exactas, Universidad de Guanajuato, Guanajuato, Mexico; ^3^Trayectoria en Genómica Alimentaria, Universidad de la Ciénega del Estado de Michoacán de Ocampo, Sahuayo, Mexico

**Keywords:** plant defensins, inflammatory response, MAPKs, HDACs, mammary epithelium

## Abstract

Defensins are an important group of host defense peptides. They have immunomodulatory properties, which have been mainly described for mammal defensins, but similar effects for plant defensins remain unknown. Previously, we showed that the defensin γ-thionin (*Capsicum chinense*) reduces *Staphylococcus aureus* internalization into bovine mammary epithelial cells (bMECs) while inducing Toll-like receptor 2 (TLR2), modulating the inflammatory response. Here, we analyze the effect of γ-thionin on the TLR2 pathway in bMECs infected with *S. aureus* and determine if it modulates epigenetic marks. Pre-treated bMECs with γ-thionin (100 ng/ml) reduced the basal activation of p38 and ERK1/2 (~3-fold), but JNK was increased (~1.5-fold). Also, infected bMECs induced p38, but this effect was reversed by γ-thionin, whereas ERK1/2 was reduced by infection but stimulated by γ-thionin. Likewise, γ-thionin reduced the activation of Akt kinase ~50%. Furthermore, γ-thionin induced the activation of transcriptional factors of inflammatory response, highlighting EGR, E2F-1, AP-1, and MEF, which were turned off by bacteria. Also, γ-thionin induced the activation of histone deacetylases (HDACs, ~4-fold) at 24 h in infected bMECs and reduced LSD1 demethylase (HDMs, ~30%) activity. Altogether, these results demonstrated the first time that a plant defensin interferes with inflammatory signaling pathways in mammalian cells.

## Introduction

Defensins constitute one of the largest groups of host defense peptides (also named antimicrobial peptides), present throughout evolution, in fungi, plants, as well as in invertebrates and vertebrates. These peptides are involved in the first line of defense of the innate immune response (IIR) against pathogens. In mammals, there are different types of defensins, which can be classified according to their genomic organization, cysteine spacing, and intramolecular disulfide connectivity (1). These are β-defensins (which are considered the ancestral type), α-defensins, and θ-defensins. In particular, β-defensins have been widely studied for their role in preventing the colonization of microbes such as bacteria, viruses, parasites, and fungi on different epithelial surfaces (2).

Besides their antimicrobial activity, β-defensins have recognized immunomodulatory properties such as the regulation of pro- and anti-inflammatory responses through affecting signaling pathways, recruiting effector cells such as phagocytes, enhancing extracellular and intracellular bacterial killing, promoting immune cell maturation, and differentiation modulating wound repair, among others (2, 3).

Many of these proinflammatory effects occur via defensin–receptor binding, and it appears that defensins can interact with a variety of immune receptors such as chemokine or Toll-like receptors (1). In addition, some of the immunomodulatory effects of β-defensins can be achieved by interfering with MAPK signaling pathways (4).

On the other hand, plant defensins belong to a large and diverse evolutionary group of proteins called the cis-defensin superfamily (5), which were considered as evolutionarily unrelated with α-defensins and β-defensins from mammals. However, recent evidence suggests that they might originate from a common ancestor via an amino acid deletion mutation in the structural motif (6), keeping their original function: the antimicrobial activity.

Thus, far, the immunomodulatory properties of plant defensins have not widely been studied. Evidence from our group shows that the defensin γ-thionin from *Capsicum chinense*, is able to modulate elements of the IIR of bovine mammary epithelial cells (bMECs) infected with *Staphylococcus aureus*, which constitutes an *in vitro* model for studying bovine mastitis (7). Using this model, we demonstrated that γ-thionin reduced the internalization of *S. aureus* into bMECs while inducing the expression and membrane abundance of Toll-like receptor 2 (TLR2), as well as induced the expression of genes coding for the pro-inflammatory cytokines TNF-α and IL-1β. γ-Thionin also induced the expression of the mRNA of anti-inflammatory cytokine IL-10 (~12-fold). Interestingly, this defensin also induced the production of nitric oxide (NO) and the secretion of the endogenous antimicrobial peptide DEFB1 by bMECs (7). In addition, the induction of epigenetic modifications has been reported in bMECs, such as H3 acetylation (8). Besides, recently Kweh et al. (9) have reported that β-defensin expression in bMECs is likely influenced by DNA methylation and histone acetylation. However, we do not know if similar modifications could be achieved by γ-thionin in bMECs.

Considering that γ-thionin induces TLR2 expression in bMCEs, in the present work, we analyze if this peptide could also interfere with signaling pathways related to this receptor, in the same way as the analogous mammalian defensins, and if these effects could be related to epigenetic modifications.

## Materials and Methods

### Peptide

The γ-thionin used in this research corresponds to the mature region (NH_2_-QNNICKTTSKHFKGLCFADSKCRKVCIQEDKFEDGHCSKLQRKCLCTKNC-COOH) of the chemically synthesized peptide (Genbank AF128239) obtained from Invitrogen. The formation of disulfide bonds was accomplished by air oxidation in 5% (v/v) aqueous dimethyl sulfoxide and was corroborated as previously reported (7). For all of the experiments, a final concentration of vehicle DMSO 0.02% was employed (control). γ-Thionin was used at 100 ng/ml for 24 h in agreement with reports describing this concentration as immunomodulatory in bMECs (7).

### *S. aureus* Strain

The *S. aureus* subsp. *aureus* (ATCC 27543) strain was used, which was isolated from a case of bovine clinical mastitis with the capacity to invade bMECs (10). *S. aureus* was grown at 37°C overnight in Luria-Bertani broth (LB Bioxon), and the CFUs were adjusted by measuring the optical density at 600 nm (OD 0.2 = 9.2 × 10^7^ CFU/ml).

### Primary Culture of bMECs

bMECs were isolated from the alveolar tissue of the udders of healthy lactating cows (slaughtered for meat production) as described (10), which were obtained at the slaughterhouse. This protocol was approved by the ethical committee for animal welfare from Universidad Michoacana de San Nicolás de Hidalgo. Cells from passages two to eight were used in all of the experiments. bMECs were cultured in growth medium (GM) composed by DMEM medium/nutrient mixture F12 Ham (DMEM/F12K, Sigma) and supplemented with 10% fetal calf serum (Equitech Bio), 10 μg/ml insulin (Sigma), 5 μg/ml hydrocortisone (Sigma), 100 U/ml penicillin, 100 μg/ml streptomycin, and 1 μg/ml amphotericin B (Invitrogen). bMECs were grown in a 5% CO_2_ atmosphere at 37°C.

### Experimental Design

In this work, the effect of the pre-treatment of bMECs with γ-thionin was analyzed with or without the infection with *S. aureus*. Prior to the infection, bMECs were incubated with γ-thionin (100 ng/ml) in DMEM/F12K (Sigma) without antibiotics and serum for 24 h. The infection assays were performed using gentamicin protection assays as described in the following section, and bMECs were infected for 2 h (10). bMECs treated with vehicle (DMSO 0.02%) were employed as control in all of the experiments. For the RNA, nuclear protein, or total protein extractions, bMECs were processed according to the protocols described below. For flow cytometry analysis of MAPK activation, total protein was obtained from cell lysates, and for AKT analysis, flow cytometry was performed in whole cells (see below). Enzymatic activity of HDACs was determined in cultured bMECs, whereas HDM activity was measured in cell lysates.

### Infection Assays

bMECs polarized monolayers were created on plates coated with 6–10 μg/cm^2^ rat-tail type I collagen (Sigma). The invasion assays were performed using gentamicin protection assays as described (10). Briefly, ~10,000 cells were cultured onto 96-well flat-bottom dishes (Corning), which were incubated with the vehicle (DMSO, 0.02%) or with 100 ng/ml of γ-thionin in DMEM/F12K without antibiotics and serum for 24 h and then were infected with *S. aureus* (MOI 30:1 bacteria per cell). For this, bMECs were first counted with a hemocytometer and then were inoculated with the corresponding volume of bacterial suspension to 9.2 × 10^7^ CFU/ml and incubated for 2 h in 5% CO_2_ at 37°C. Then, bMECs were washed three times with PBS (pH 7.4) and cultured in GM without serum and penicillin and streptomycin, but supplemented with 80 μg/ml gentamicin for 1 h at 37°C to eliminate extracellular bacteria. The last step was achieved with the purpose to only analyze the effect of internalized bacteria.

### MAPK Activation

To evaluate the MAPK activation levels by flow cytometry, the bMECs were treated with 100 ng/ml γ-thionin (24 h), *S. aureus*, or both, and 30 μg of protein of the samples were prepared according to the manufacturer's protocol for adherent cells (Becton Dickinson, Germany). Briefly, denaturation buffer (Becton Dickinson) was added to cultured bMECs in 24-well plates, in the presence of protease inhibitor cocktail (Sigma) and 4% IGEPAL (Sigma). Then, bMECs were scraped from the wells and transferred to 1.5-ml tubes. Samples were denatured in a boiling water bath for 5 min. Then, DNA was sheared from the samples by sonication. Protein concentrations were determined with Bradford assay. pp38 (T180/Y182), pJNK1/2 (T183/185), and pERK1/2 (T202/Y204) were quantitatively determined in bMEC lysates using antibodies from a Flex Set Cytometric Bead Array (CBA, Becton Dickinson) according to the manufacturer's protocol. This array has been developed to analyze MAPKs (or any other analyte) using a series of particles with discrete fluorescence intensities to simultaneously detect multiple soluble proteins from serum, plasma, or tissue culture lysates (as in the present work) or supernatant samples. This assay is a particle-based immunoassay (beads), which consists in specific capture beads that are mixed with recombinant protein standards or test samples, then incubated with phycoerythrin (PE)-conjugated detection antibodies to form sandwich complexes. The flow cytometry analyses were performed using the BD Accuri^TM^ C6 and CBA analysis FCAP software (Becton Dickinson). A total of 3,000 events (beads) were acquired following the supplied protocol (https://www.bdbiosciences.com). The minimum detection levels for each phospho-protein were 0.38 U/ml for pJNK and 0.64 U/ml for pp38 and pERK. The FCAP Array software enables complete analysis of data files and extrapolation of sample values (bMECs lysates) by comparison against a standard curve for each phospho-protein. The negative controls were bMECs treated with pharmacological inhibitors of p38 (5 μM, SB203580), JNK (20 μM, SP600125), or ERK1/2 (2.5 μM, U0126) for 30 min.

### Transcription Factor–DNA Interactions

Nuclear proteins were obtained from the bMECs. Briefly, bMECs were washed 3× in phosphate-buffered saline (PBS), lysed on ice for 10 min in extraction buffer A [10 mM HEPES; pH 7.9, 10 mM KCl, 10 mM EDTA, protease inhibitor cocktail (Sigma), and 4% IGEPAL] with shaking. bMECs were harvested from the wells by scraping into the extraction buffer and pipetting to disrupt cell clumps. Nuclei were collected by centrifugation 12,000 × rpm for 3 min. The pellet was then resuspended in 200 μl of extraction buffer B (20 mM HEPES, pH 7.9, 400 mM NaCl, 1 mM EDTA, 10% glycerol, protease inhibitor cocktail, and 10 mM DTT) and incubated for 2 h at 4°C. The nuclear extracts were then centrifuged for 5 min at 15,000 × *g* and the supernatant was collected. Protein concentrations were determined with the Bradford assay. The nuclear extracts were subjected to the TranSignal Protein/DNA array I (Panomics, Fremont, CA, USA), according to the manufacturer's protocol without modifications. Briefly, biotin-labeled DNA-binding oligonucleotides (TranSignalTM Probe Mix) were incubated with 15 μg/ml of nuclear extracts to allow the formation of the transcription factor (TF)/DNA complexes as previously described (11). These complexes were separated from free probes (using the columns provided in the kit), and they were hybridized to the Panomics protein/DNA array I, consisting of 56 TFs related with inflammatory response. Finally, the complexes were detected using an HRP-based chemiluminescence method according to the manufacturer's protocol. The DNA samples are spotted in duplicate in two rows on the array (top: undiluted; bottom: dilution 1/10). Positive control consisting of biotinylated DNA was spotted for alignment along the right and bottom sides of the array. The National Institute of Health ImageJ program was used to determine the optical density [arbitrary units (AU)] of spots on the array, which was expressed as the ratio of the optical density of the TF/optical density of the DNA control.

### Western Blot Analysis of FAK

Confluent cells were treated with γ-thionin (100 ng/ml) for 24 h. After treatment, cells were infected with *S. aureus* (ATCC 27543) for 2 h and total proteins were obtained using RIPA buffer (150 mM NaCl, 1% NP-40, 0.5% sodium deoxycolate, 0.1% SDS, and 50 mM Tris–HCl, pH 8). Protein concentration was measured by Bradford method, and 20–30 μg of each sample was resolved on 10% SDS-PAGE. Proteins were transferred to PVDF membranes and then were blocked with 5% non-fat milk in PBS for 4 h at 4°C. After that, membranes were incubated with primary antibodies at 4°C overnight: anti-FAK (Santa Cruz SC-1688) 1:250 and anti-phospho FAK Y397 (Santa Cruz SC-81493) 1:250. Anti-GAPDH (Santa Cruz SC-47724) 1:500 was used as loading control. Membranes were incubated with a mouse secondary antibody horseradish peroxidase-conjugated (Thermo Scientific 7076S) 1:2,500 for 4 h at room temperature. Finally, ECL Western blotting substrate (Pierce, Thermo Scientific) was added and membranes were exposed to X-ray film. Signal intensity was quantified by densitometry using the National Institute of Health ImageJ program.

### AKT Activation

The bMECs were cultured in 24-well dishes (Corning) and were treated with γ-thionin (100 ng/ml) for 24 h and/or *S. aureus* as described above. After the treatments, the cells were washed three times with PBS, detached with trypsin (0.05%) and EDTA (0.02%), centrifuged at 2,500 rpm for 10 min at 4°C, and washed with PBS. The bMEC pellet was fixed with fixation buffer (BD Cytofix; BD Biosciences) for 15 min at 37°C, and then permeabilized with perm wash buffer (Perm Buffer III, BD Biosciences) for 25 min at 37°C. Then, bMECs were blocked with normal goat serum (5% in PBS, Pierce) for 30 min at 4°C with shaking. After that, bMECs were incubated again with Perm Buffer III for 25 min at 37°C. Finally, the cells were centrifuged and the pellet was incubated with 5 μl of the monoclonal phycoerythrin (PE)-coupled anti-pAkt antibody (pS473, BD Biosciences-560378) for 1 h at 4°C with shaking in the dark to a final volume of 100 μl in Perm Buffer III, and finally washed three times with PBS and resuspended in 100 μl of PBS. The fluorescent signals of 10,000 events were measured using the BD Accuri^TM^ C6 cytometer and analyzed with the FlowJo software.

### Histone Deacetylase (HDAC) Activity Assay

bMECs (1 × 10^5^) were grown in plates of 96 wells with γ-thionin (100 ng/ml) in serum and antibiotic-free medium during 6, 12, or 24 h, and then were infected with *S. aureus* for 2 h. HDAC activity was measured using Fluor-de-Lys® kit (Enzo LifeSciences) to analyze class I HDACs (HDAC1, 2, 3, and 8), IIa (HDAC4, 5, 7 and 9), and IIb (HDAC6 and 10). Then, bMECs were incubated with 2,000 pmol of the acetylated Fluor-de-Lys® substrate for 4 h. Cell, media, and standard curve of deacetylated substrate were prepared according to the manufacturer's directions; relative fluorescence unit (RFU) counts were acquired with a Varioskan (Thermo Scientific) plate reader (360 nm excitation, 460 nm emission). A standard curve was generated using serial 1:10 dilutions of the deacetylated Fluor-de-Lys standard and developer supplied with the BML-AK503 HDAC fluorometric cellular activity assay kit. Trichostatin (TSA 1 μM) was purchased from Sigma and was used as an inhibitor of HDACs. HDAC activity was calculated considering the effect of vehicle as basal activity (normalized to 1-fold).

### Histone Demethylase (HDM) Activity Assay

We employed a kit from ThermoFisher (Cat. EIAHDMF) to measure the activity of LSD1 and Jumonji family demethylases (HDMs) in bMECs treated with the peptide γ-thionin and infected. Cells were grown in plates of 96 wells and treated as described for HDAC assays. Cell lysates were obtained with 25 μl of the Tris-based buffer for cell lysis provided with the kit. Then, we added the enzymatic reaction mix (provided with the kit) containing 25 μl of JMJD2A (Jumonji family enzyme) to 50 μl of a 2 mM ascorbate, 100 μM FeSO_4_ solution, and 25 μl of a specific sequence of Histone H3 with the trimethylated lysine at amino acid 9 (H3K9me3) containing 2 mM alpha-ketoglutarate plus all cofactors and inhibitors dissolved in Jumonji-type Assay Buffer. The plate was incubated 1 h at 30°C. Finally, 25 μl of formaldehyde reagent was added into each well. Plates were incubated for 30 min at 37°C. A standard curve with formaldehyde was running in parallel. Demethylase reaction final volume was 100 μl in each well. The product of the enzymatic demethylation reactions is formaldehyde, which was quantitated directly by a fluorescent product with a Varioskan (Thermo Scientific) plate reader (450 nm excitation, 510 nm emission). HDM activity was calculated considering the effect of vehicle as basal activity (normalized to 1-fold).

### Statistical Analysis

The data were analyzed with the PRISM8.0 software, performing a one-way analysis of variance (ANOVA) using the *post-hoc* Tukey test (*P* ≤ 0.05 was considered statistically significant), with the exception of (1) the densitometric analysis for TF differentially expressed, which was analyzed using multiple *t*-test analysis considering values of *P* ≤ 0.05 as statistically significant, and (2) the HDM activity assay, which was analyzed with *t*-test (*P* ≤ 0.05) because two different enzymes are shown in the graph. For ANOVA, different letters indicate differences among treatments. For the experiments of pFAK, pAKT, HDACs, and HDMs, the values were normalized considering the effect on vehicle-treated bMECs as 1 (fold induction). The number of replicates for the different experiments is indicated in each figure legend; for the assays of enzyme activity, duplicates were performed according to the manufacturer's instructions.

## Results

### Regulation of MAPK by γ-Thionin in bMECs

**Figure 1:**
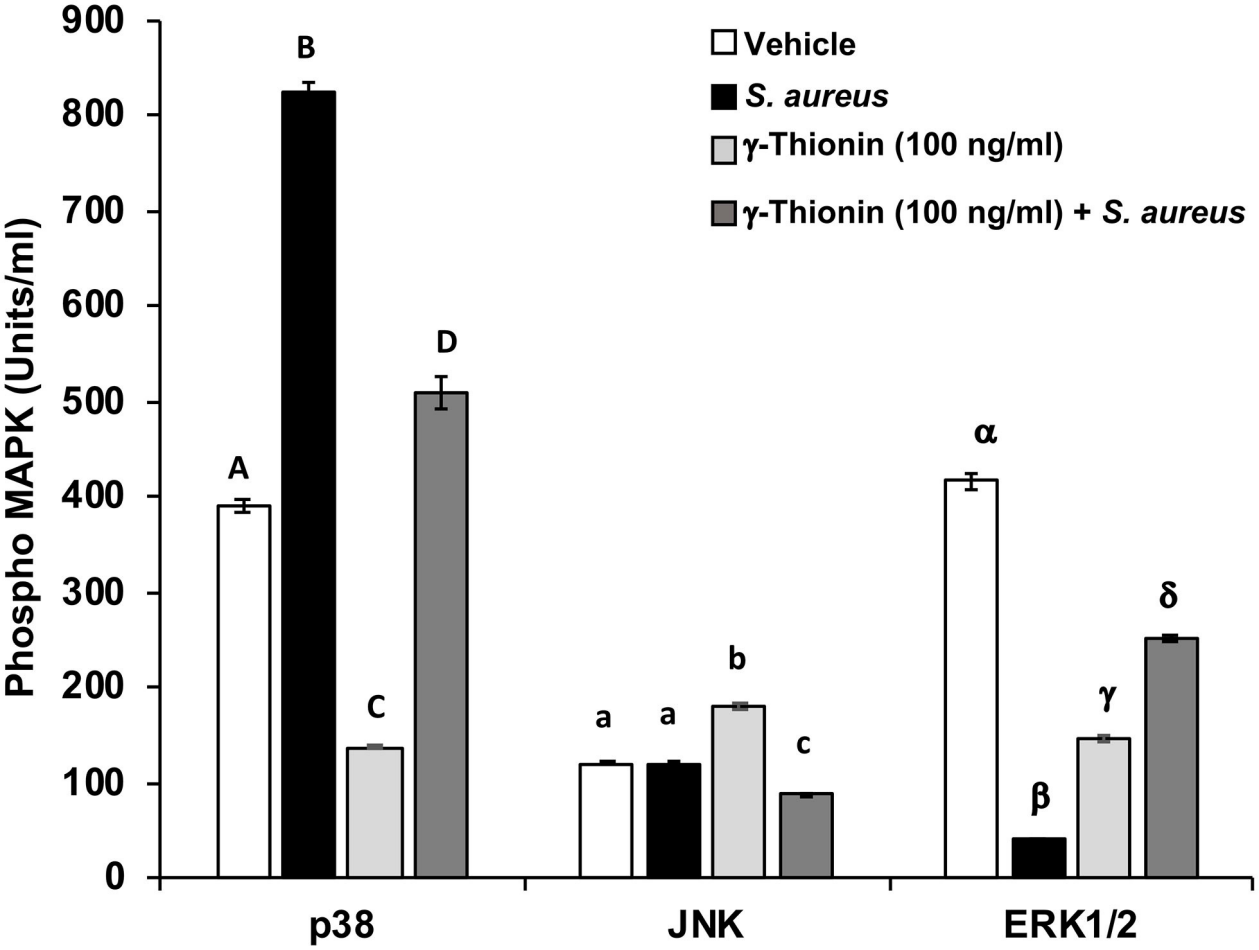
p38, JNK, and ERK1/2 activation regulated by γ-thionin in bMECs. MAPK phosphorylation was measured in bMECs treated with γ-thionin (100 ng/ml) by flow cytometry. Each bar shows the mean ± SD of 3,000 events acquired using a Flex Set Cytometric Bead Array (CBA, Becton Dickinson). Data were obtained from two different experiments, which were run in duplicate (*n* = 4). Different letters between each MAPK analyzed indicate significant differences (one-way ANOVA, *post-hoc* Tukey test *P* ≤ 0.05), within the treatments (capital letters for p38, lowercase letters for JNK, and Greek symbols for ERK1/2). Vehicle was DMSO 0.02%.

In a previous work, we demonstrated that γ-thionin at 100 ng/ml reduces *S. aureus* internalization into bMECs and is able to activate TLR2. Because TLR2 activation leads to MAPK phosphorylation (p38, JNK, or ERK1/2), which are involved in bacteria phagocytosis, we analyzed the phosphorylation status of these MAPKs in γ-thionin-treated bMECs. The p38 phosphorylation level was increased (~2-fold) in *S. aureus*-challenged bMECs, but the activation of ERK1/2 was reduced (~2-fold) and the JNK activation was not modified (Figure 1). When bMECs were treated with γ-thionin (100 ng/ml, 24 h) the basal activation of p38 and ERK1/2 was reduced (~3-fold for both), but the activity of JNK was increased (~1.5-fold) (Figure 1). In the presence of *S. aureus*, the pre-treatment with γ-thionin maintained reduced levels of p38 in relation to bacteria alone (~1.6-fold), as well as the levels of JNK. However, the activation of ERK1/2 was induced in the presence of *S. aureus* in bMECs pre-treated with the peptide (~6.2-fold) (Figure 1). In the presence of MAPK inhibitors, the levels of MAPK phosphorylation in bMECs were reduced as follows: 54.29 pg/ml for JNK (65%), 23.675 pg/ml for ERK 1/2 (94%), and 3.335 pg/ml for p38 (92%).

### Activation of FAK and Akt

**Figure 2:**
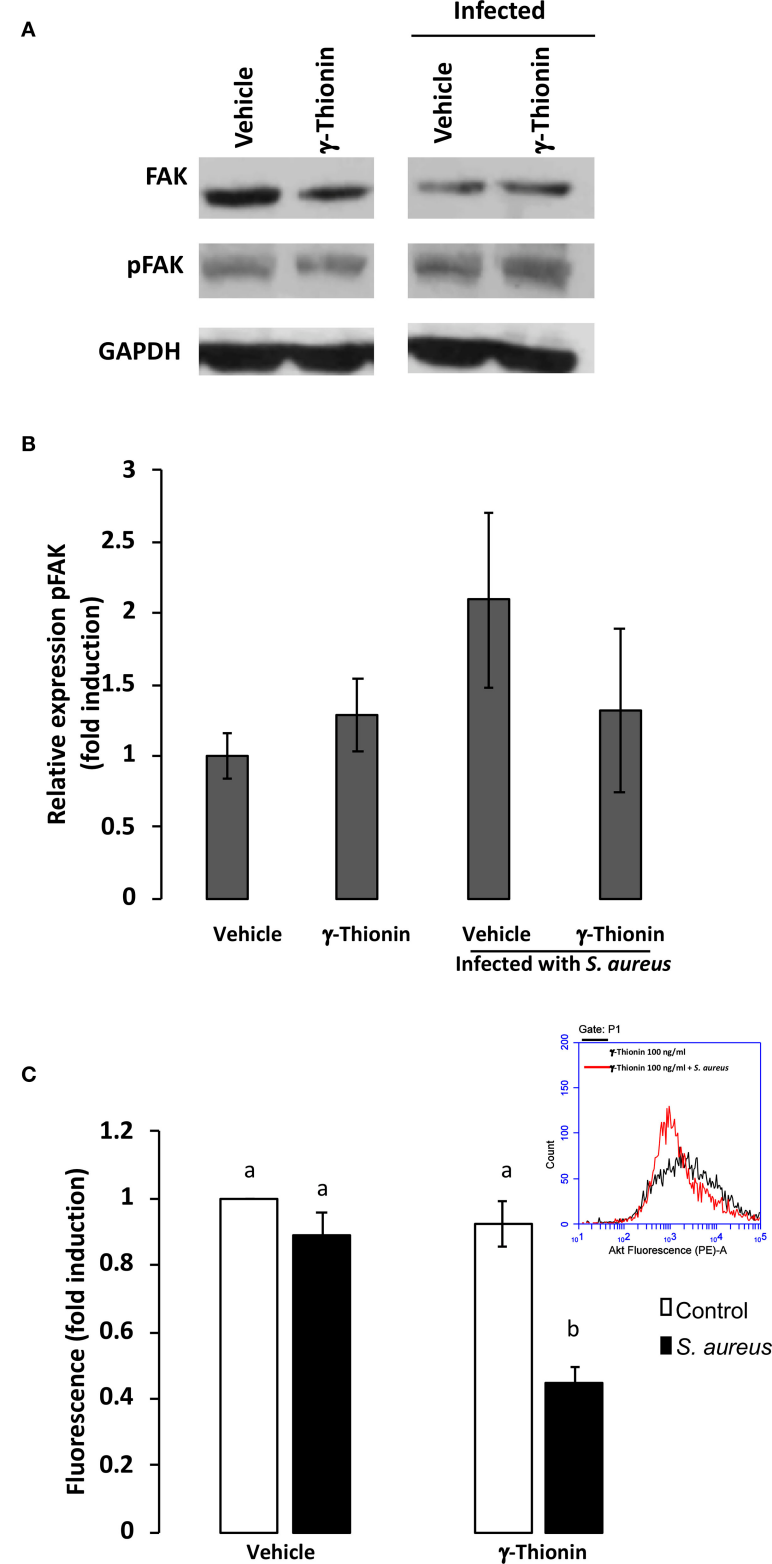
AKT is down-regulated by γ-thionin and *S. aureus* in bMECs. **(A)** Western blot analysis from cell lysates of bMECs treated 24 h with γ-thionin (100 ng/ml) with or without *S. aureus* infection for 2 h. Lysates were immunoblotted for total-FAK, phospho-FAK, or GAPDH as control. DMSO 0.02% was used as vehicle. **(B)** Densitometrical analysis of the immunoblots that shows the relative expression of pFAK with respect to total-FAK. Each bar shows the mean ± SD of optical density (arbitrary units, AU) considering the expression of control cells (vehicle, DMSO 0.02%) as 1 (data normalized), from three different experiments (*n* = 3). **(C)** The relative fluorescence intensities of AKT activation (phospho-AKT) in bMECs treated with γ-thionin (100 ng/ml) and infected with *S. aureus* are shown. Fluorescence intensity was estimated from 10,000 events. An inserted histogram plot that shows AKT staining data in γ-thionin-treated bMECs (black line) and cells treated with the peptide that were challenged with *S. aureus* (red line). Each bar shows the mean of fluorescence of 10,000 events ± *SD*, considering the fluorescence of control bMECs as 1 (data normalized). Data were obtained from two different experiments, which were run in triplicate (*n* = 6). Different letters between each condition analyzed indicate significant differences (one-way ANOVA, *post-hoc* Tukey test *P* ≤ 0.05) within the treatments. Vehicle was DMSO 0.02%.

It has been shown that FAK is involved in the TLR2-mediated inflammatory response (12) and in *S. aureus* internalization (13). Thus, we analyzed this kinase in bMECs treated with γ-thionin. We did not detect changes in FAK phosphorylation in bMECs treated with γ-thionin or in the presence of *S. aureus* (Figures 2A,B). In order to explore if other kinases related to FAK pathway could participate in this effect, we also determined the activation of protein kinase B (Akt), detecting a reduction in its phosphorylation state in γ-thionin pre-treated bMECs and infected with *S. aureus* (Figure 2C).

### Activation of TFs Related to the IIR

**Figure 3:**
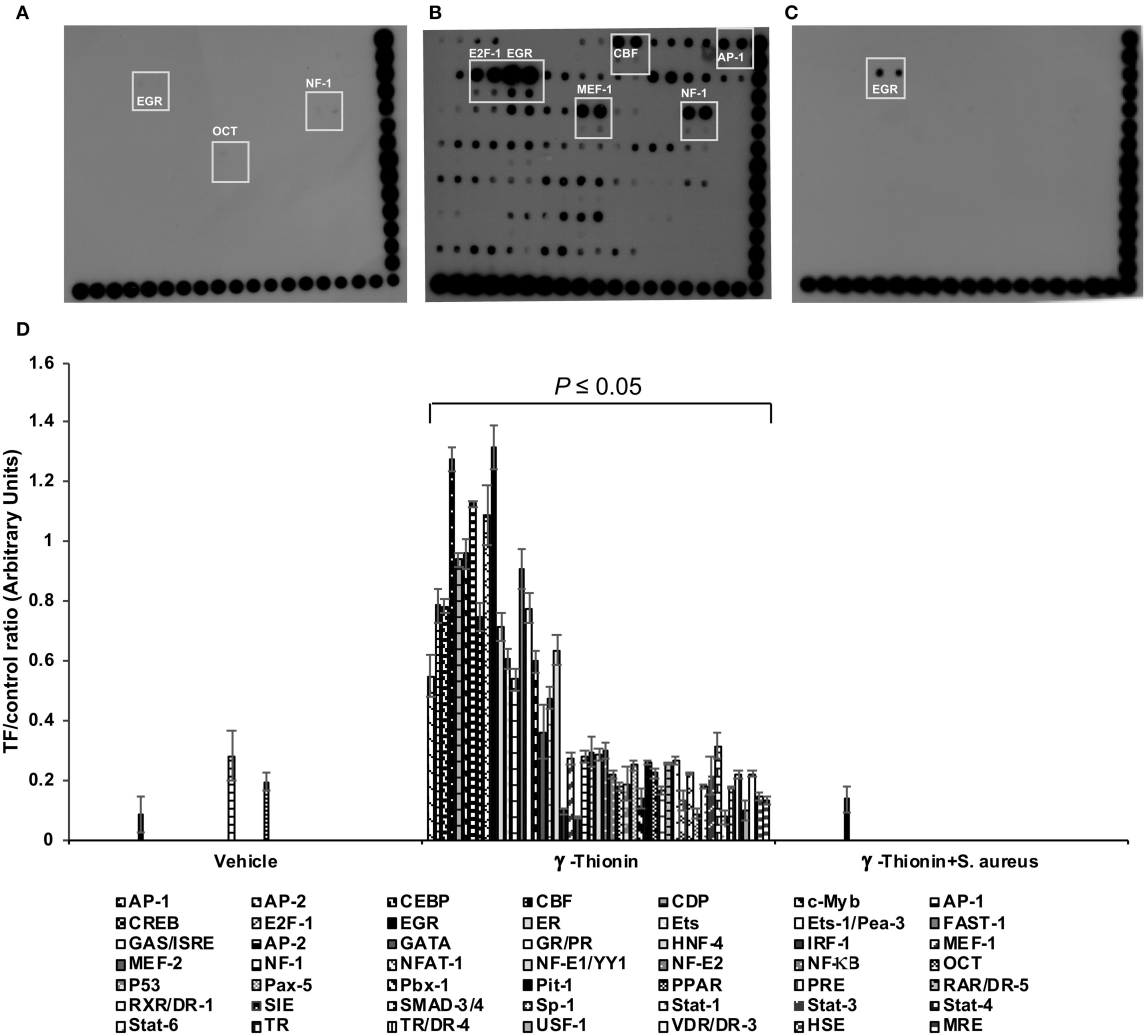
Transcription factor activation by γ-thionin in bMECs infected with *S. aureus*. Protein/DNA array blots were used to analyze 56 different transcription factor DNA-binding sites from samples that were obtained from **(A)** bMEC nuclear extracts treated with DMSO 0.02% (vehicle, control), **(B)** bMECs that were treated with 100 ng/ml of γ-thionin for 24 h, and **(C)** bMECs that were treated with 100 ng/ml of γ-thionin for 24 h and infected with *S. aureus* for 2 h. **(D)** The bars show the mean intensity ± SD of duplicates of the ratio TF/control [arbitrary units (AU) of optical density]. Multiple *t*-test analysis was performed considering values of *P* ≤ 0.05 as statistically significant in relation to bMECs treated with vehicle (DMSO, 0.02%). The intensity of each spot from the membranes was quantified using ImageJ software. The DNA samples were spotted in duplicate in two rows (top: undiluted; bottom: dilution 1/10). Biotinylated DNA (control) was spotted for alignment along the right and bottom sides of the array. The TFs that were activated by all of the treatments are highlighted with boxes. AP-1, activating-protein 1; CBF, core binding factor; EGR-1, early growth response protein 1; E2F-1, E2F transcription factor 1; MEF-1, myeloid Elf-1 like factor; NF-1, nuclear factor 1; OCT-1, octamer transcription factor.

In bMECs, TLR2 activation triggers signaling pathways that induce the activation of transcriptional factors (TFs) related to host defense, such as AP-1, NF-κB, E2F-1, etc. (14–16). Previously, we demonstrated that γ-thionin (100 ng/ml) induces the expression of pro- and anti-inflammatory cytokines in bMECs, as well as TLR2 activation, but the TFs activated by the peptide remained unknown. Thus, we evaluated the activation status of 56 TFs related to IIR using a protein/DNA array (Figure 3). The bMECs treated with vehicle exhibited a null activation of TFs (Figure 3A). After 24 h of γ-thionin treatment, we detected the activation of 46 different TFs related to inflammatory response, highlighting the activation of E2F-1, EGR, CBF, AP-1, MEF, and NF-1. Interestingly, after *S. aureus* challenge in γ-thionin pre-treated bMECs, only EGR displayed an evident signal (Figure 3C). A densitometrical analysis of the differentially expressed TFs is showed in Figure 3D. These results suggest that γ-thionin induces the expression of elements of the IIR in bMECs through the activation of different TFs, which are down-regulated in the presence of *S. aureus*.

### Activity of HDACs and HDMs

**Figure 4:**
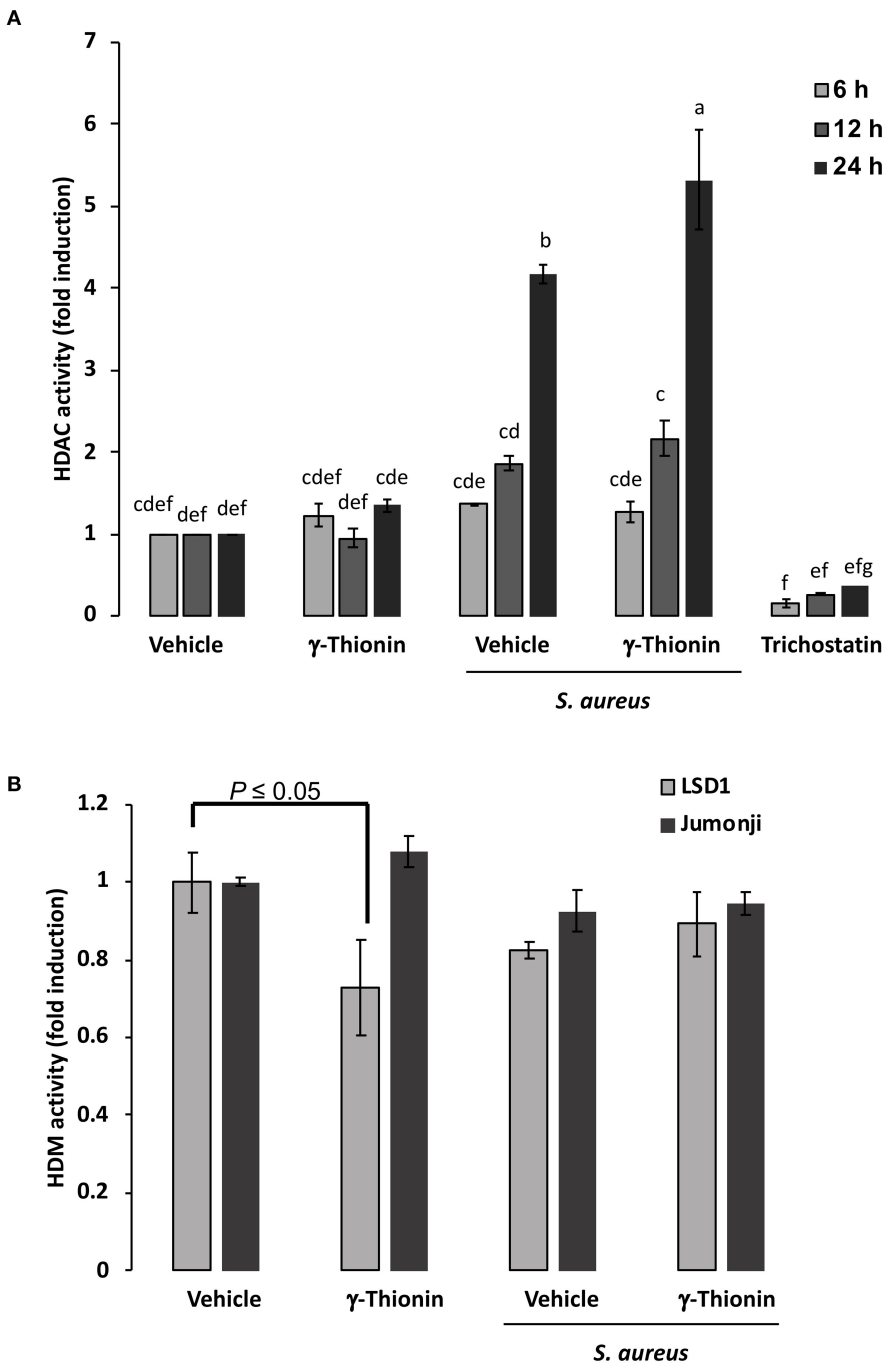
HDAC and HDM activation in bMECs treated with γ-thionin and infected with *S. aureus*. bMEC lysates were obtained at 6, 12, or 24 h of treatment with γ-thionin (100 ng/ml) with or without 2 h of *S. aureus* infection and were incubated with **(A)** the substrate for Class I and II HDACs accordingly to manufacturer's instructions. Total HDAC enzyme activity was determined by using the HDAC fluorometric cellular activity assay Fluor de Lys®. Each bar shows the mean of HDAC activity of cells from two different experiments ± SD, which were run in duplicate (*n* = 4), considering the expression of control cells (vehicle, DMSO 0.02%) as 1 (data normalized). Different letters indicate significant differences between each condition (one-way ANOVA, *post-hoc* Tukey test *P* ≤ 0.05); Trichostatin (1 μM) was used as an inhibitor of HDCAs. In **(B)**, bMEC lysates were obtained at 24 h of treatment with γ-thionin (100 ng/ml) with or without 2 h of *S. aureus* infection and were incubated with the substrates for LSD1 and Jumonji demethylases (HDMs). Each bar shows the mean of HDM activity of cell lysates from two different experiments ± *SD*, which were run in duplicate (*n* = 4), considering the expression of control cells (vehicle, DMSO 0.02%) as 1 (data normalized). *t*-test (*P* ≤ 0.05) was performed between treatments for the same HDM.

To explore if γ-thionin could achieve its effects through the regulation of epigenetic modifications, we tested the activity of HDACs using a cellular histone deacetylase fluorescent assay kit, which measures the activity of different class I and class II HDACs. According to results, infection induced the activation of HDACs (Figure 4A, ~2-fold), which was enhanced in 12- and 24-h γ-thionin-treated bMECs (~2- and 4-fold, respectively), suggesting that this peptide could be involved in modifications of the chromatin. Interestingly, this effect was not detected in bMECs treated only with the peptide. On the other hand, results indicate that in bMECs treated 24 h with γ-thionin, the activity of HDM LSD1 was significantly reduced (~0.3-fold, Figure 4B), suggesting that the levels of histone methylation are maintained under the treatment with the peptide.

## Discussion

In the present work, we analyzed the effect of γ-thionin on the TLR2 pathway in bMECs infected with *S. aureus*. Previously, we have demonstrated that this peptide is able to increase the membrane abundance of TLR2 in bMECs treated during 24 h with 100 ng/ml γ-thionin (7). Under these conditions, internalization of *S. aureus* into bMECs is reduced and the expression and secretion of different cytokines are modulated. Considering that TLR2 is necessary for the first interaction of bMECs with *S. aureus*, in this work we explore the TLR2 signaling pathways triggered by γ-thionin. Thus, we first analyzed the activation of different MAPKs. Previous results from our group indicate that MAPKs (p38, JNK, and ERK1/2) are required for *S. aureus* internalization into bMECs, because their blockage reduces bacterial uptake (14). However, this requirement does not necessarily involve kinases being activated. According to results, γ-thionin reduced the basal activation of p38 and ERK1/2 (~3-fold), whereas JNK activity was increased (~1.5-fold). Also, infected bMECs induced p38 (~2-fold); this effect was slightly reduced by γ-thionin, but it remained significantly activated in relation to control cells (1.3-fold). In this sense, Wang et al. (17) have reported that p38 is induced by *S. aureus* in bMECs, whereas work from our group shows that only JNK is activated by *S. aureus* (11). Thus, the reduction in p38 activation could be related to the reduction in internalization detected in bMECs treated with γ-thionin (7) because this kinase can be activated by FAK (12). In agreement with this, we have previously demonstrated that internalization of *S. aureus* is inhibited in bMECs treated with a selective p38 inhibitor (SB203580, ~50%), indicating the role of this kinase during bacterial internalization (14). Given that in this work we did not detect changes in the levels of FAK phosphorylation, we suggest that other mechanisms should be implicated with p38 activation. In that respect, the reduction in Akt activation induced in bMECs treated with γ-thionin and infected can be involved with the bacterial internalization reduction produced by the peptide. In agreement with this hypothesis, Oviedo-Boyso et al. (18) have reported that *S. aureus* induces a time-dependent Akt activation in bovine endothelial cells infected with the same bacterial strain employed in the present work. Also, they showed that Akt activation decreases at longer infection times (1–2 h), coinciding with our results. Thus, the reduction on p38 and Akt activation induced by γ-thionin during *S. aureus* infection of bMECs can be related to the decrease in internalization (7). In this work, we did not detect JNK activation by *S. aureus*. Other report from our group indicates that *S. aureus* can activate JNK in bMECs cultured in GM without serum and antibiotics. In this work, we performed all the assays in GM treated with DMSO (0.02%); therefore, the discrepancy between these results and the previous report can be attributable to the vehicle employed (14). In addition, JNK activation has been demonstrated upon *S. aureus* infection in different models (19, 20). In this work, we also shown that ERK1/2 activity was reduced by infection (~10-fold) but stimulated by γ-thionin (~6-fold). These results agree with previous reports from our group (11). However, Wang et al. (17) have reported that ERK is lightly activated during infection of bMECs with *S. aureus* using a MOI of 1:1, which can explain the differences. Considering these results, we suggest that ERK1/2 is not necessary for *S. aureus* internalization into bMECs; however, it can be important for the modulation of the inflammatory response of bMECs during *S. aureus* infection previously reported (7). In order to determine the possible transcriptional factors activated by γ-thionin, we performed a microarray of 56 different TFs related to inflammatory response. Our results indicate that the TFs E2F-1, EGR, CBF, AP-1, MEF, and NF-1 are highly activated by the peptide, suggesting that the IIR genes regulated by γ-thionin in bMECs, such as TLR2, TNF-α, IL-1β, IL-6, and IL-10 (7), could be activated through these pathways. However, we detected that in bMECs treated with γ-thionin and infected with *S. aureus*, EGR is the only transcriptional factor activated, whereas the others remained turned off. These results can be related to the enhanced pro-inflammatory response that we have described in bMECs infected and treated with 100 ng/ml of γ-thionin, which is mainly associated with the membrane abundance of TLR2, and the secretion of TNF-α, IL-6, and the antimicrobial peptide DEFB1 (7). Considering that in the present model, the MAPKs ERK1/2 and p38 are activated in bMECs in the presence of *S. aureus* and the peptide, the pathways TLR2/ERK/EGR and/or TLR2/p38/EGR could be responsible for this enhanced pro-inflammatory response. EGRs comprise a gene family of transcriptional factors related to different functions. In particular, EGR-1, which is contained in the microarray employed, has been related to growth, development, differentiation, apoptosis, fibrosis, and inflammation (21).

In the present work, we also show that γ-thionin (100 ng/ml) induces, in a time-dependent manner, the activation of HDACs in infected bMECs, showing the highest effect at 24 h of treatment. Deacetylation, mediated by HDACs, favors tight binding of DNA to the histones, thus favoring compact chromatin, which is related to prevention of gene transcription (22). More than 18 different HDACs have been identified to date based on their phylogenetic analyses and sequence homologies: Class I HDACs (HDACs 1, 2, 3, and 8), class II HDACs (HDACs 4, 5, 6, 7, 9, and 10), class IV HDAC (HDAC 11), and class III HDACs that consist of sirtuins (23). In the present work, we employed a kit that measures the activity of class I and class II HDACs. The HDAC activation in bMECs treated 24 h with γ-thionin and infected with *S. aureus* coincides with the inhibition in the activation of most of the TFs related to inflammation. Thus, γ-thionin could modulate the inflammatory response through epigenetic modifications, which is a novel effect for this kind of molecules. The role of HDACs in the regulation of inflammatory mediators such as cytokines and chemokines in different diseases including infections has been widely documented; in fact, HDAC inhibitors represent a therapeutic target for many inflammatory conditions (23). In agreement with the results obtained, HDAC activation in bMECs treated with γ-thionin and infected could be the consequence of the activation of p38 and/or ERK1/2. It is worthy to highlight that the induction in HDAC activity coincides with the activity of p38 in infected bMECs alone or treated with γ-thionin. Accordingly, recent work from Romanick et al. (24) has shown that HDAC1/2 inhibition in a bMEC line (MAC-T cells) attenuated JNK and ERK activation and, thus, inflammatory gene expression. However, in the present work, the specific molecular target(s) that can be affected by γ-thionin in bMECs remains to be elucidated. Furthermore, the previous results related to HDAC activity are in agreement with the reduced activity of HDM LSD1 in bMECs treated 24 h with γ-thionin (Figure 4B), suggesting that the levels of histone methylation are maintained in bMECs treated with the peptide, favoring therefore the tight binding of DNA to the histones. However, the target genes epigenetically modulated by γ-thionin remain unknown and require further research.

## Conclusion

In this work, we show that γ-thionin induces MAPKs in infected bMECs, demonstrating that plant defensins can interfere with inflammatory signal transduction pathways in mammals. γ-Thionin also reduces the activation of FAK and Akt kinases, which could be related to the reduction of *S. aureus* internalization into bMECs. γ-Thionin effects on bMECs also involve epigenetic modulation, comprising a novel property for this kind of molecules. The inflammatory target genes epigenetically modulated by γ-thionin require further research.

## Data Availability Statement

The raw data supporting the conclusions of this article will be made available by the authors, without undue reservation, to any qualified researcher.

## Ethics Statement

The protocol to obtain the primary culture of bovine mammary epithelial cells was approved by the ethical committee for animal welfare from Universidad Michoacana de San Nicolás de Hidalgo.

## Author Contributions

MB-M, NA-M, JL-M, and AO-Z designed the experiments. MB-M, MA-M, and IM-E performed the experiments. MB-M, AO-Z, and JL-M analyzed the data. JL-M and AO-Z wrote the paper. All authors contributed to the article and approved the submitted version.

## Conflict of Interest

The authors declare that the research was conducted in the absence of any commercial or financial relationships that could be construed as a potential conflict of interest.
